# A program to train medical physicists for direct patient care responsibilities

**DOI:** 10.1002/acm2.12472

**Published:** 2018-10-17

**Authors:** Derek W. Brown, Todd F. Atwood, Kevin L. Moore, Robert MacAulay, James D. Murphy, Arno J. Mundt, Todd Pawlicki

**Affiliations:** ^1^ Dept of Radiation Medicine and Applied Sciences UC San Diego Moores Cancer Center La Jolla CA USA; ^2^ Professional Development Center School of Medicine UC San Diego La Jolla CA USA

**Keywords:** clinical communication skills, direct patient care, medical physicists, training program

## Abstract

**Objectives:**

To develop a training program designed to meet the specific needs of medical physicists as they transition into a clinical role with direct patient care responsibilities.

**Materials and Methods:**

The training program was designed in collaboration with the faculty at the UC San Diego School of Medicine and incorporates training techniques that have been shown to be effective in improving communication skills. The program emphasizes experiential, practice‐based learning over didactic presentations.

**Results:**

The training program is comprised of 5 components: 1) a 1‐day Clinician‐Patient Communication Workshop run by the UC San Diego School of Medicine, 2) Communication Strategies for Radiation Oncology, which consists of two, 2‐hour sessions designed to provide trainees with patient communication skills that are specific to patient interactions in radiation oncology, 3) Simulated Patient Interactions, in which trainees perform mock physicist‐patient consults with trained patient actors, 4) Faculty‐Observed Patient Consults, and 5) a Case‐Based Treatment Toxicity Course. A competency assessment mechanism was also developed to provide a clear set of objectives and to guide trainer feedback. [Correction added after first online publication on November 7, 2018: The phrase ", which consists of two, 2‐hour" was added above.]

**Conclusions:**

The training program that we have developed incorporates an array of established education techniques and provides a comprehensive, accessible, means of improving medical physicists’ patient communication skills.

## 1 INTRODUCTION

We recently proposed, and demonstrated the efficacy of, a new role for medical physicists that includes having them develop independent, professional relationships with patients.^1^, ^2^ This initiative addresses a demonstrated shortcoming in the provision of treatment‐related information by healthcare professionals in the field of radiation oncology.^3^, ^4^, ^5^ Given that inadequate communication is known to lead to reduced adherence to treatment regimes^6^ and increased likelihood of medical error,^7^ this initiative also addresses the patient safety hazard that this shortcoming presents.

As technical experts, medical physicists are uniquely positioned to be able to guide patients through the treatment process, to address questions about treatment delivery and imaging modalities, as well as to discuss concerns about radiation safety. In contrast to the extensive training received by physicians,^8^, ^9^ there are no comparable resources for medical physicists.^10^, ^11^ Moreover, we are not aware of any independent training programs that are specifically designed to teach medical physicists to communicate effectively with patients.

Here, we describe a training program that was developed to meet the specific needs of medical physicists as they transition into a clinical role with direct patient care responsibilities.

## 2 STRUCTURE OF THE TRAINING PROGRAM

The training program incorporates established techniques for improving patient communication skills. It emphasizes experiential, practice‐based learning over didactic presentations and includes role‐playing and Simulated Patient interactions.^12^, ^13^, ^14^, ^15^ The five steps are described below.

### 2.1 ClinicianPatient Communication Workshop

The medical physics training program utilizes a Physician Assessment and Clinical Education Workshop which is a 1‐day event offered through the UC San Diego School of Medicine. It is designed specifically to improve physician–patient communication and employs a structured approach to training communication skills based on four main concepts — engagement, empathy, education, and enlistment.^16^

### 2.2 Communication strategies for radiation oncology

This course consists of a series of two, 2‐hour sessions designed to provide trainees with patient communication skills that are specific to patient interactions in radiation oncology. The sessions combine brief lectures with moderated, practice‐based group interactions.

Topics covered are:

1. The essential components of a physicist–patient consult.
2. How to introduce yourself to a patient.
3. Review of a list of common questions from patients.
4. Strategies for answering patient questions effectively.
5. Re‐enactments of positive and negative physicist–patient interactions followed by group discussion.
6. A one‐on‐one role‐playing exercise where each physicist plays the role of a patient during an encounter with a medical physicist.

### 2.3 Simulated Patient Interactions

Widely used in medical education, Simulated Patient interactions have been shown to be effective in improving clinical communication skills.^17^ Working in collaboration with the Professional Development Center at the UC San Diego School of Medicine, we developed two simulated patient models. One, was for a tech‐savvy and curious breast cancer patient, and the other, for a non‐technical and nervous prostate cancer patient. Two Simulated Patients were hired and trained to play the roles of the patients in physicist–patient consults.

Simulated Patient characteristics and demeanors are withheld from trainees before the simulated physicist–patient consult. An important aspect of this exercise is for the trainees to practice assessing a patient's informational needs and comfort level with technical language while using patient‐appropriate language throughout the consult.

Trainees perform consults with both Simulated Patients. Each consult is scheduled for 30 minutes. A trainer is present in the room during the consult to assess the trainee's clinical communication competencies using the form shown in Table 1. Both trainers and Simulated Patients provide verbal feedback to the trainees immediately after each consult and consults are videotaped, reviewed, and discussed with trainees.

**Table 1:** Clinical communication competency assessment form for physicist–patient consults

Physicist–Patient consult communication competency assessment	Score
1. How well does the trainee introduce themselves and describe their role in the clinic?	
2. How well does the trainee explain that they are the primary resource for all technical aspects related to the patient's treatment?	
3. How well does the trainee provide a basic overview of the entire radiation therapy process?	
4. How well does the trainee explain the purpose of the CT simulation appointment?	
5. How well does the trainee determine whether or not the patient will have difficulty during the CT simulation?	
6. How well does the trainee assess the patient's comfort level with radiation therapy and answer any questions they have about the treatment process?	
7. How well does the trainee describe the treatment planning process and explain how the patient's personalized plan was created?	
8. How well does the trainee describe the treatment delivery process and explain how the linear accelerator delivers the treatment?	
9. How well does the trainee respond to questions related to the treatment planning or treatment delivery process?	

### 2.4 Faculty‐Observed Patient Consults

Trainees conduct actual physicist–patient consults with a medical physicist mentor present in the room. The medical physicist mentor assesses the trainee's clinical communication competencies using the same clinical communication competency form. Trainees meet with the medical physicist mentor prior to, and immediately after, the consult to discuss the interaction.

### 2.5 Case‐Based Treatment Toxicity Course

Medical faculty in our department run a case‐based course that describes common treatment toxicities. Classes occur once per month over the course of a year and cover common toxicities for breast, genitourinary, gynecological, brain, pediatrics, lymphoma, head and neck, gastrointestinal, and lung cases. Toxicity presentation, progression, and likelihood of resolution are discussed. Medical physicist trainees take this course for the purpose of equipping them with the knowledge to better prepare for understanding likely medical questions from patients and to help them effectively communicate toxicity concerns to the attending radiation oncologist.

**Figure 1:**
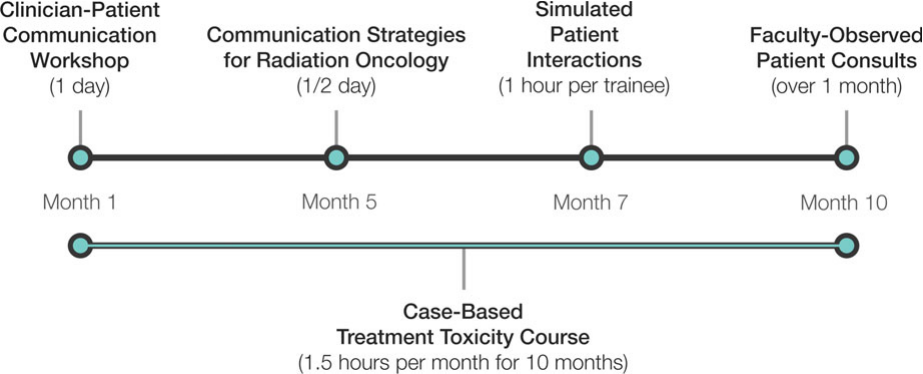
Schematic timeline of the training program as implemented thus far. The Clinician‐Patient Communication Workshop and the Simluated Patient Interactions are done in collaboration with the School of Medicine. [Correction added after first online publication on November 7, 2018: Wording of previous sentence was changed.]

Figure 1 provides a schematic timeline of the program.

## 3 COMPETENCY ASSESSMENT

A series of clinical communication competencies, shown in Table 1, were developed to reflect the primary goals of patient interactions. For example, in our experience, many patients arrive for their CT simulation appointment with limited understanding of the purpose of the appointment. One of the primary goals for such an interaction that occurs immediately prior to CT simulation, therefore, is for the physicist to provide a basic overview of the radiation therapy process and to explain the specific purpose of the CT simulation appointment. Another observation is that patients often have concerns about the use of radiation in their treatment. This is reflected in a specific competency assessment that asks if the physicist is able to assess the patient's comfort level with radiation therapy and answer any questions they may have.

Clinical communication competencies are assessed at two time points: (a) during the Simulated Patient interactions, and (b) during the Faculty‐Observed Patient Consults. A three‐point scoring system was developed for this assessment, defined as follows:

1. Trainee did not attempt to display the specific competency.
2. Trainee attempted to display the specific competency but missed some key elements.
3. Trainee displayed mastery of the specific competency.

Trainees are deemed competent to perform physicist–patient consults on their own when they score a three in all competency categories.

## 4 INCORPORATION OF DIRECT PATIENT CARE TRAINING IN MEDICAL PHYSICS RESIDENCY PROGRAMS

If direct patient care responsibilities for medical physicists are to see widespread adoption, appropriate training programs will need to be implemented in medical physics residency programs. This raises several important questions: (a) How will educators prepare for developing and running such a training program?, (b) How much clinical experience should a resident have prior to starting the training?, and (c) Is there sufficient time in a 24‐month residency to incorporate this type of training?

To address the first question, we propose to offer a series of “train‐the‐trainer” workshops where residency directors would acquire the experience and materials required to implement a similar training program in their own residency programs. Ultimately this work could be taken over by relevant committees within the AAPM. In response to the second question, we believe that training should begin as early as possible. Given there is sufficient oversight and that the competency assessment described in this work is adhered to, there is little risk of putting residents in situations where they would have insufficient clinical experience to successfully interact with patients. With respect to whether there is sufficient time in a 24‐month residency program, we believe that this new role for medical physicists is of sufficient importance that time must be made to ensure adequate training of residents. Perhaps programs could reduce the amount of time spent doing monthly, or patient‐specific, QA to make time for this type of training.

## 5 SUMMARY

The concept of training medical physicists to be patient‐facing professionals is new to the field of radiation oncology. The training program that we have developed incorporates an array of established education techniques and provides a comprehensive, accessible, means of improving medical physicists’ patient communication skills. It was created in conjunction with a phase II clinical trial, recently conducted at our institution, where board certified medical physicists established independent professional relationships with patients. The results of that phase II trial showed decreases in patient anxiety and increases in patient satisfaction.^2^

Additional trainees are currently going through the program in preparation for a prospective randomized phase III clinical trial. This cohort of trainees will complete the training program before the end of 2018. A formal evaluation of the program is ongoing and will be submitted for peer review after the current trainees have completed the program and are routinely participating in physicist–patient consults.

## CONFLICT OF INTEREST

The authors have no other relevant conflicts of interest to disclose.
